# A new technique for measurement of subrotational lifetime of molecular ions

**DOI:** 10.1038/s41598-020-77408-0

**Published:** 2020-11-20

**Authors:** Jyoti Rajput, Herendra Kumar, Pragya Bhatt, C. P. Safvan

**Affiliations:** 1 Department of Physics and Astrophysics, University of Delhi, Delhi, 110007 India; 2 Inter-University Accelerator Centre, Aruna Asaf Ali Marg, New Delhi, 110067 India

**Keywords:** Atomic and molecular physics, Physical chemistry

## Abstract

Singly and multiply charged molecular ions are found in diverse environments and hold relevance for a wide range of research areas like combustion chemistry, accelerator physics, atmospheric sciences, plasma physics, astrophysics etc. Molecular dications are of special significance as they can be generated and studied comparatively easily in laboratory experiments. And they have enabled exploration of new and exciting phenomenon such as hydrogen migration, inter-atomic Coulombic decay, plasmonic excitations, orbital tomography etc. The lifetime of a molecular dication is one of its fundamental characteristics, whose measurement contributes to strengthening *ab initio* calculations and in predicting the concentration of its dissociation products. Most of the already reported lifetimes of molecular dications are in the range of nanoseconds to seconds and metastable states with lifetimes of the order of picoseconds have only been theoretical predicted and an experimental verification is pending. We present a method of measuring subrotational lifetimes of molecular dications formed in three-body sequential breakup of polyatomic molecular precursors. Specifically, we have measured the subrotational lifetime of $\hbox {SO}^{2+}$ , which is formed as an intermediate in the three-body sequential fragmentation of $\hbox {SO}_2^{3+}$. The lifetime against dissociation is determined to be a fraction of the rotational period of $\hbox {SO}^{2+}$ and is of the order of few picoseconds. The method proposed is general and is not restricted to triatomic precursors.

## Introduction

Spontaneously dissociating (unstable) and metastable molecular ions provide insights into a wide variety of physical phenomena and serve as sensitive test cases for theoretical approaches. Specifically, the determination of the lifetime of molecular dications has been a topic of fundamental interest and experimental measurements have been carried out since the time it became technologically feasible^1^. Such studies are also significant in understanding and modeling low density plasmas under terrestrial, planetary and other astrophysical conditions^2^(and references therein). Several of the simpler molecular dications like that of carbon monoxide ($\hbox {CO}^{2+}$)^3,4^ and and nitrogen ($\hbox {N}_2^{2+}$)^5,6^ have already been extensively studied and the lifetimes of many of their metastable states are known today. The lifetimes of molecular dications have also been a subject of several *ab initio* calculations^4,7–10^ which report values in a very wide range from picoseconds to seconds. On the other hand experimental measurements of these lifetimes report values in the range of several nanoseconds to few milliseconds or higher. Lifetime measurements in the range of microseconds to seconds have been traditionally carried out using storage rings^11–13^ and translational energy spectroscopy^14^. With the advent of multi-particle coincident time-of-flight measurements, lifetimes for a variety of molecular ions were reported^15–17^ primarily in the range of nanoseconds. The experimental confirmation of existence of molecular dicationic states having lifetimes in few picoseconds is an open research problem. Lifetimes in the very short time scales though difficult to access experimentally provide stringent tests for theoretical calculations since the effects of the breakdown of Born–Oppenheimer approximation and electron correlations would be clearly visible. Present measurements on extremely short temporal evolutions which make use of attosecond and femtosecond pulses of laser/synchrotron radiation are not directly applicable for multiply charged ions.

In this article we present an experimental method to determine the subrotational lifetime of a molecular dication, when such an ion is formed during a molecular dissociation process. Thus we address the natural decay of a dication in the absence of any external photon field and without the use of high resolution time-resolved techniques like femtosecond pump probe. For this we consider the sequential three-body breakup of a triply charge molecular ion, $\hbox {SO}_2^{3+}$. We obtain the lifetime of $\hbox {SO}^{2+}$, which is formed as a molecular intermediate in the three-body sequential breakup of $\hbox {SO}_2^{3+}$ leading to detection of ($\hbox {O}^+$+$\hbox {O}^+$+$\hbox {S}^+$). The process considered is the following:

$$\begin{aligned} \begin{array}{lcccc} \hbox {Step 1:} &{} ~ &{} \hbox {SO}_2^{3+} &{} \rightarrow &{} [\hbox {SO}^{2+}] + \hbox {O}^{+} \\ ~ &{} ~ &{} ~ &{} ~ &{} ~ \\ \hbox {Step 2:} &{} ~ &{} [\hbox {SO}^{2+}] &{} \rightarrow &{} \hbox {S}^+ + \hbox {O}^{+} \end{array} \end{aligned}$$

The three-body breakup of a molecular ion requires cleavage of two bonds. If this cleavage happens within the same single vibration of the two bonds, it’s called concerted breakup and if there is a finite time delay between the breaking of the two bonds then it’s termed as sequential. Several previous investigations focusing on sequential and concerted three-body breakup using simple linear molecules like $\hbox {CO}_2$^18,19^, OCS^20–23^, $\hbox {CS}_2$^24,25^, $\hbox {N}_2$O^26^ and $\hbox {C}_2 \hbox {H}_2$^27^ and different means of initiating the ionisation (leading to dissociation) have been reported. Since the sequential as well as concerted pathways of breakup yield the same set of final three fragment ions, separating the data based on the primary pathway has attracted much attention. It is to be noted that in the sequential mechanism of breakup a metastable molecular intermediate is generated during the first step (e.g. $\hbox {SO}^{2+}$ as shown above). At present, three broad representations are employed for addressing the two breakup mechanisms: the Dalitz plot^18,28^, the Newton diagram^18^ and the native frame approach^20^ of data analysis.

**Figure 1:**
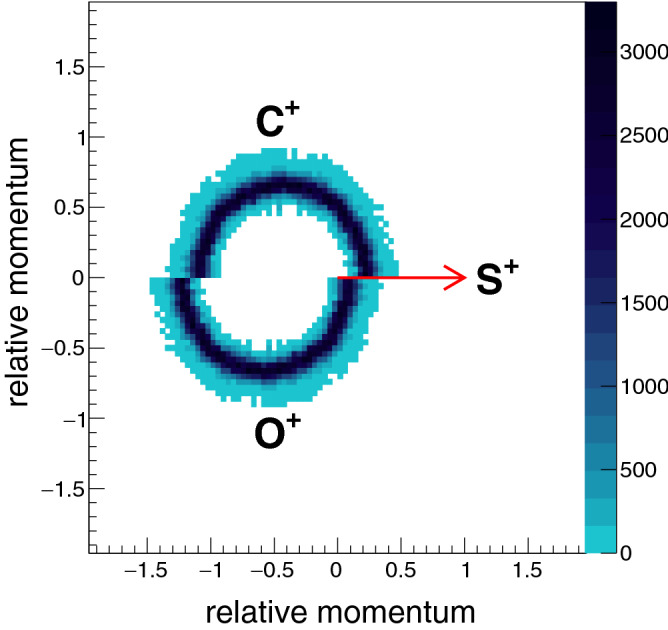
Newton diagram for three-body sequential breakup of a model system, $\hbox {OCS}^{3+}$ via the $\hbox {CO}^{2+}$ intermediate. The momentum of the $S^{+}$ ion is taken as reference for normalisation and set as a unit vector along the x-axis and a distribution of components of the other two normalized momenta [$\frac{\vec {p}_C}{|\vec {p}_S|}$,$\frac{\vec {p}_O}{|\vec {p}_S|}$] are projected in the upper and lower half plane.

**Figure 2:**
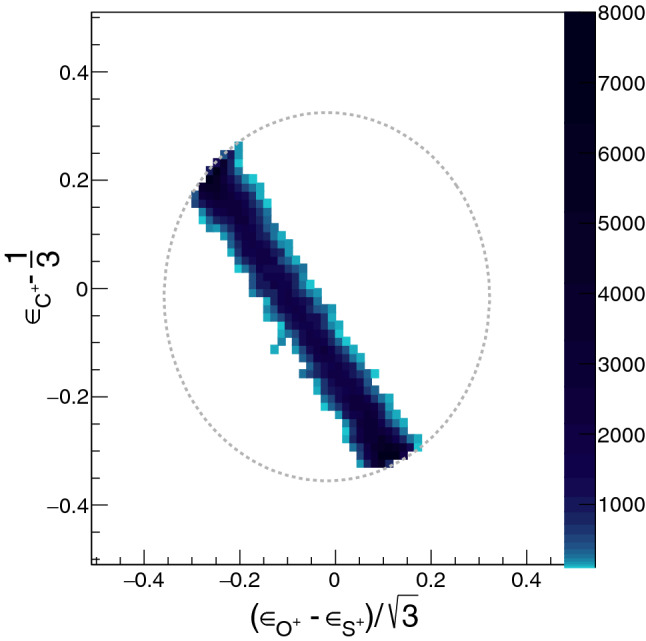
Dalitz plot for three-body sequential breakup of a model system, $\hbox {OCS}^{3+}$ via the $\hbox {CO}^{2+}$ intermediate. $\epsilon _{i} = \frac{|\vec {p_i}|^2}{\Sigma |\vec {p_i}|^2}$ where i = $\hbox {C}^+$, $\hbox {O}^+$, $\hbox {S}^+$. Momentum and energy conservation restricts all events to lie within the dotted circle.

All these three representations use the correlated momenta of the three fragment ions measured in coincidence. To highlight the features of a sequential three-body breakup as seen in the Dalitz plot and Newton diagram, we have simulated the process using a heteronuclear model system $\hbox {OCS}^{3+}$. The two steps of the breakup are (i) $\hbox {OCS}^{3+} \rightarrow [\hbox {CO}^{2+}] + \hbox {S}^{+}$ (ii) [$\hbox {CO}^{2+}$] $\rightarrow$ $\hbox {C}^+ + \hbox {O}^{+}$. The simulation employs the Coulomb explosion model to generate momenta of the three dissociation fragments ($\hbox {C}^{+}$, $\hbox {O}^{+}$, $\hbox {S}^{+}$) and the resulting Newton diagram and Dalitz plot are shown in Figs. 1 and 2 respectively. The Coulomb explosion model assumes a unit point charge being placed on each atom of the constituent molecular ion and considers the Coulomb repulsion between these ions leading to fragmentation. Thus the finite span of the electron cloud is not taken into account and only $\frac{1}{r}$ dependence of the potential is considered.

For the Newton diagram, we have assumed that the lifetime of the intermediate $\hbox {CO}^{2+}$ is much greater than its rotational time period (an assumption based on reported results^20,23^). And hence, the angle between the momenta of fragments generated from the first and the second step can have any value leading to semicircular arc like distribution as seen in Fig. 1.

A Dalitz plot is a representation of the energy correlation between the various fragments. The energy and momentum conservation demands that all events on a Dalitz plot be confined within a circular region. In Fig. 2, which shows the Dalitz plot for the three-body sequential breakup of $\hbox {OCS}^{3+}$, the band like feature is a signature of the sequential process. Usually only part of the band is clearly observed in actual experimental data due to overlap with the dominant concerted breakup channel.

The broad observations from the studies on the separation of events based on concerted and sequential breakup mechanisms, over the past two decades can be summarised in the following three points: (1)a large difference between the energy of the terminal fragment ions is most likely associated with the sequential mode of breakup as evidenced also from the Dalitz^18,28^ plot generated for three-body dissociation processes. (2) the appearance of a semicircular arc like feature in the Newton diagram^18^ is a signature of the presence of sequential mode of breakup. (3) the probability of a sequential breakup is usually much lower (< 20%) as compared to that of the concerted breakup.

In the native frames approach^20^,the three-body sequential dissociation of a triply charged molecule is analysed in two different frames of reference, one being the centre-of-mass frame of the triply charged ion (step 1) and the other being the centre-of-mass frame of the doubly charge molecular intermediate (step 2).

In our experiment, we have collected coincident data for three-body breakup of $\hbox {SO}_2^{3+}$ into ($\hbox {O}^+ + \hbox {O}^+ + \hbox {S}^+$) and analysed it by first using a Dalitz plot^18,28^ to select a particular set of events which have a high probability of coming from the sequential mode of breakup of $\hbox {SO}_2^{3+}$ (via $\hbox {SO}^{2+}$ intermediate), followed by applying the native frames approach^20^ on the selected set. As an additional check on the selected set of events, a Newton diagram is also plotted which confirms the origin of these events as arising from sequential breakup. This allowed us to get a direct estimate of the lifetime ($\tau$) of intermediate $\hbox {SO}^{2+}$ molecular ion in terms of its rotational period ($\hbox {T}_R$). Assuming a value of 16 ps for the rotational period ($\hbox {T}_R$) of $\hbox {SO}^{2+}$ ion and the experimentally determined value of the ratio ($\tau$/$\hbox {T}_R$), we have estimated the lifetime ($\tau$) of its metastable state to be $\approx$2.0 ps.

## Experimental details

The experiment was conducted at the Low Energy Ion Beam Facility (LEIBF) of Inter-University Accelerator Centre, New Delhi. Projectile ions of $\hbox {Xe}^{9+}$, produced in an electron cyclotron resonance ion source placed on a high voltage deck, accelerated to an energy of 450 keV were made to interact with neutral $\hbox {SO}_2$ molecules (effusing from a hypodermic needle) under single collision conditions^29^. The recoil ions generated from this interaction were extracted into a time-of-flight mass spectrometer using an electric field of 50 V/mm while the electrons are extracted in the opposite direction. The recoil ions formed are detected by a micro channel plate based position sensitive detector equipped with a delay line that allows us to record the two dimensional position as well as the time of arrival of the recoil ion. Our data acquisition system with its multi-hit time-to-digital convertor allows for detection of all fragments in coincidence. From the measured time and position information of individual ions, and the known geometry of the time of flight spectrometer and knowledge of the applied electric fields, the correlated momentum vectors of all ions originating from every single dissociation event are extracted. The methodology used is similar to the one described by Dorner et al.^30^. For every dissociation event of an original $\hbox {SO}_2$ molecule, all fragment ions are captured in coincidence. Neutral fragments, which may be formed during molecular dissociation, are not detected in our experiment.

## Results and discussion

**Figure 3:**
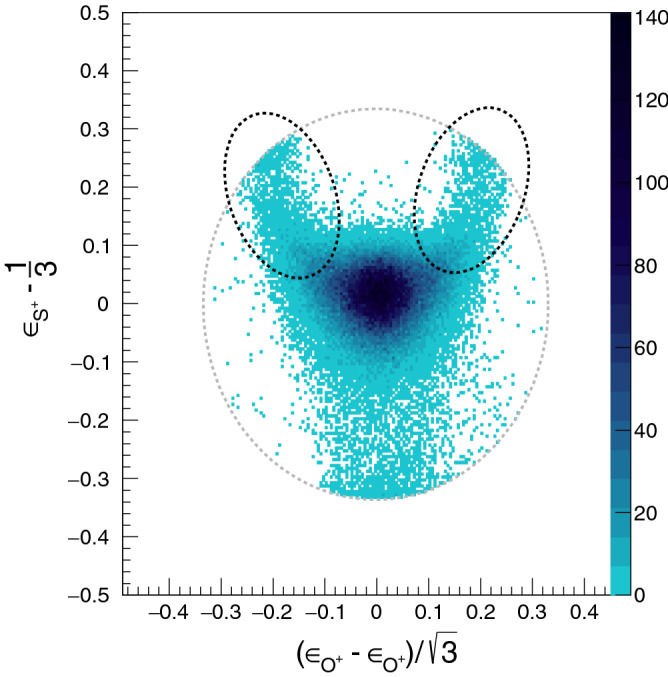
Dalitz plot for $\hbox {SO}_2^{3+}$ dissociating into ($\hbox {O}^++\hbox {O}^++\hbox {S}^+$). $\epsilon _{i} = \frac{p_i^2}{\Sigma p_i^2}$ where $p_i$ is the magnitude of momentum of the *i*th fragment. The events within the black elliptical regions have a large energy difference between the two identical $\hbox {O}^{+}$ fragment ions and are attributed to the sequential mode of breakup of $\hbox {SO}_2^{3+}$. Momentum and energy conservation restricts all events to lie within the dotted grey circle.

Figure 3 shows the Dalitz plot generated using the experimentally measured momenta of the ions ($\hbox {O}^+$, $\hbox {O}^+$, $\hbox {S}^+$) formed in the three-body breakup of $\hbox {SO}_2^{3+}$. The events within the black elliptical regions are attributed to the sequential mode of breakup of the parent ion $\hbox {SO}_2^{3+}$ via the $\hbox {SO}^{2+}$ intermediate. These regions correspond to the case when the energy difference between the two identical $\hbox {O}^{+}$ fragment ions is large. We determined the magnitude of the energy difference between the two $\hbox {O}^+$ fragment ions for events within the selected elliptical regions and found that for most of them the value of the difference is greater than 9.0 eV. From now on, we will work with only these selected events, which constitute only 6.1% of the total. The dark island at the centre of the Dalitz plot contains events coming predominantly from concerted three-body breakup of $\hbox {SO}_2^{3+}$ and hence not considered for the present study.

**Figure 4:**
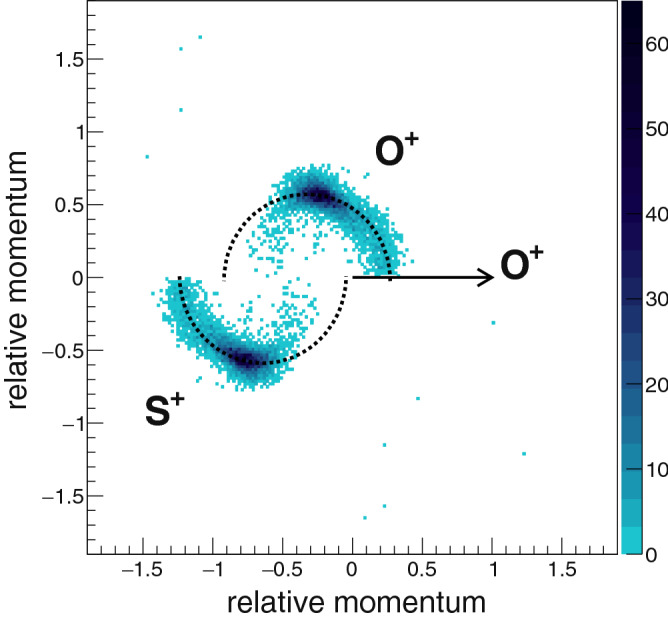
Newton diagram for $\hbox {SO}_2^{3+}$ dissociating into ($\hbox {O}^+$+$\hbox {O}^+$+$\hbox {S}^+$) for the selected set of events (see text for details). The appearance of an arc like feature signifies sequential mechanism of breakup.

While the Dalitz plot gives a good parametrization for separating events based on sequential or concerted breakup, a Newton plot gives a clearer visual impression of an active sequential process. If we make a Newton diagram for three-body breakup of $\hbox {SO}_2^{3+}$ (see Fig. 4) we see an arc like feature but not a complete semi-circle as observed for the case of three-body breakup of $\hbox {CO}_2^{3+}$^18^ and $\hbox {OCS}^{3+}$^20^. The explanation for the appearance of a semicircular arc like feature for these two molecules is the existence of a sequential mechanism for which the populated states of the molecular intermediate have a lifetime much larger than its rotational time period. We propose, that in case of sequential breakup of $\hbox {SO}_2^{3+}$ via the $\hbox {SO}^{2+}$ intermediate, the dissociation lifetime ($\tau$) of the intermediate is much smaller than its rotational time period ($\hbox {T}_R$) leading to occurrence of an arc instead of a semicircle. The high density areas on the Newton plot being at an angle with respect to the first fragment is indicative of the non-linear geometry of the parent molecular ion $\hbox {SO}_2^{3+}$. It may be pertinent to point out that the neutral $\hbox {SO}_2$ molecule has a bent geometry^31^ in its ground state, with a bond angle of 119.5$^\circ$.

**Figure 5:**
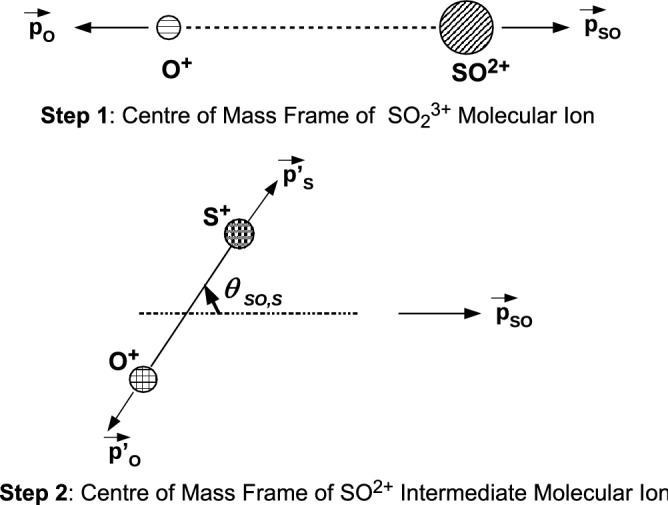
Scheme of the native frames approach for fragmentation of $\hbox {SO}_2^{3+}$ into ($\hbox {O}^{+}$+$\hbox {O}^+$+$\hbox {S}^+$) via sequential mechanism. The first step of the process is analysed in the centre-of-mass frame of $\hbox {SO}_2^{3+}$ and the second step is analysed in the centre-of-mass frame of $\hbox {SO}^{2+}$. The kinetic energy release ($\hbox {KER}_{{SO}}$) during step 2 of the breakup is determined in the centre-of-mass frame of the molecular intermediate $\hbox {SO}^{2+}$ along with the angle $\theta _{SO,S}$ which is the angle between $\vec {p}_{SO} (= \vec {p}_{O})$ and $\vec {p'}_{S}$.

**Figure 6:**
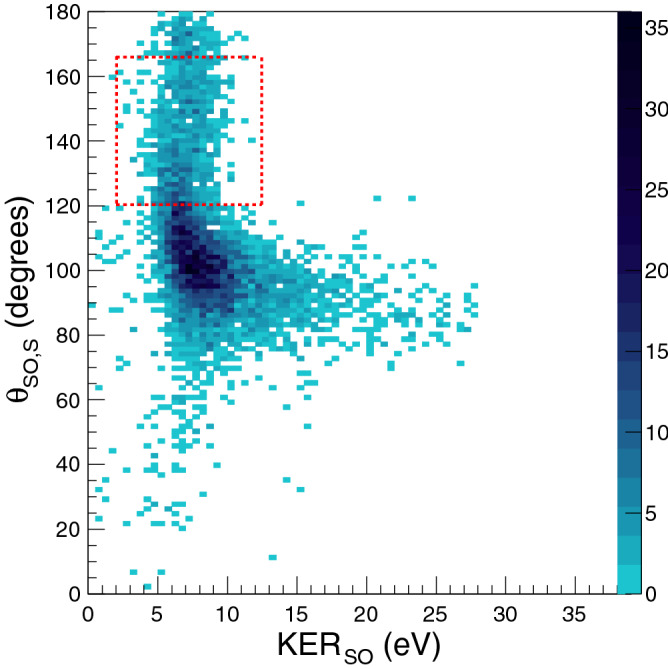
Distribution of $\hbox {KER}_{{SO}}$ and $\theta _{SO,S}$ for the selected set of experimental data. $\hbox {KER}_{{SO}}$ is the kinetic energy release in the unimolecular dissociation of SO$^{2+}$ in its centre-of-mass frame and $\theta _{SO,S}$ is the angle between $\vec {p}_{SO} (= \vec {p}_{O})$ and $\vec {p'}_{S}$. See text for details. The events within the dotted rectangle were fitted with a single exponential.

We now employ the native frames approach^20^ where in the three-body sequential dissociation of a triply charged molecule is analysed in two different frames of reference, one associated with each of the two steps of the dissociation process, see Fig. 5. Whenever an excited molecular ion dissociates into two or more fragments, the internal energy of the molecular ion gets distributed as kinetic energy between its fragments. The sum of the kinetic energy of all fragments taken together is known as the kinetic energy release (KER) of that dissociation process. Applying the native frames approach to our experiment, the first step of the process is analysed in the centre-of-mass frame of $\hbox {SO}_2^{3+}$ and the second step is analysed in the centre-of-mass frame of $\hbox {SO}^{2+}$. The kinetic energy release distribution ($\hbox {KER}_{{SO}}$) for breakup of $\hbox {SO}^{2+}$ and the angle ($\theta _{SO,S}$) between the momentum vector associated with the first step of the breakup and the internuclear axis of the molecular intermediate $\hbox {SO}^{2+}$ is determined. In Fig. 5 which shows the schematic of the breakup process, this is the angle between $\vec {p}_{SO} (= -\vec {p}_{O})$ and $\vec {p'}_{S}$. Figure 6 shows the result of using the native frames approach for the three-body breakup of $\hbox {SO}_2^{3+}$ leading to coincident detection of ($\hbox {O}^+ + \hbox {O}^+ + \hbox {S}^+$), a distribution of $\hbox {KER}_{{SO}}$ with $\theta _{SO,S}$.

For cases where the lifetime ($\tau$) of the molecular intermediate is much larger than its rotational time period ($\hbox {T}_R$), the angle between the axis of the molecular intermediate and the momentum vector associated with the first step of the breakup is expected to be uniformly distributed in the range [0,$\pi$]. This has already been shown for the case of the linear molecule OCS in two different studies^20,23^. For the case of $\hbox {SO}_2$, a molecule having a bent initial geometry, we see that the distribution of $\theta _{SO,S}$ is not uniform for the whole range. For cases where the lifetime ($\tau$) of the molecular intermediate is smaller than its rotational time period ($\hbox {T}_R$), as in the present case, a limited range of angles is expected. In addition, the maximum number of events are expected to be at an angle close to the bond angle of the parent $\hbox {SO}_2^{3+}$.

As is evident from Fig. 6, the kinetic energy $\hbox {KER}_{{SO}}$ is not constant over the angular range covered by the data. This can be explained based on a recent study on the role of a neighbor ion in the fragmentation dynamics of covalent molecules^32^ which has shown that the presence of an ionic neighbor leads to a shift in the kinetic energy release distribution (KERD) of a covalent molecule towards higher values while keeping the features of the distribution intact. In case of the sequential fragmentation of $\hbox {SO}_2^{3+}$, if the time between the first step of the breakup and the second is small, the kinetic energy of the second step of the breakup will be influenced by the presence of the $\hbox {O}^+$ formed during the first step. From Fig. 6 it is clear that the kinetic energy release for unimolecular dissociation of $\hbox {SO}^{2+}$ becomes constant (at $\approx$6.8 eV) at values of $\theta _{SO,S}$ greater than 120$^\circ$ while in the angular range 120$^\circ$ to 80$^\circ$, it is variable and higher than 6.8 eV. This can be understood in terms of the dissociation of $\hbox {SO}^{2+}$ being influenced by its partner from the first step of dissociation, $\hbox {O}^+$. As the molecular intermediate dissociates farther away from its partner, this influence decreases rapidly and the second breakup is then effectively independent of the first breakup. Only after this influence fades away, the KER for unimolecular dissociation of $\hbox {SO}^{2+}$ attains a constant value. Thus the determination of $\hbox {KER}_{{SO}}$ enables us to eliminate the events which show the influence of the nearby ion and hence focus on the natural decay of $\hbox {SO}^{2+}$ without any outside influence.

We have estimated the effect of the presence of $\hbox {O}^+$ formed during the first step of the breakup of $\hbox {SO}_2^{3+}$ on the $\hbox {KER}_{{SO}}$ of the second breakup using Coulomb model. We found that the change in the $\hbox {KER}_{{SO}}$ is less than 10%, compared to its value in the absence of any ion nearby, when the distance between $\hbox {O}^+$ is approximately 20 times the internuclear distance of $\hbox {SO}^{2+}$. For this approximation we have assumed the internuclear distance of $\hbox {SO}^{2+}$ to be a constant. With the ion-molecule interaction time of the order of sub-femtoseconds, it is reasonable to assume that the nuclear coordinates remain frozen and the initial geometry to the multiply charged molecular ion remains the same as that of the neutral precursor. Assuming the internuclear distance of $\hbox {SO}^{2+}$ to be 1.43 Å^31^, and energy of the first $\hbox {O}^+$ to be 12.5 eV (calculated using Coulomb explosion model), we found that the effect of the first fragment $\hbox {O}^+$ on the $\hbox {KER}_{{SO}}$ of the second breakup becomes negligible in less than 300 femtoseconds.

By collecting the events with $\theta _{SO,S}$ greater than $120^\circ$, we were able to fit a single exponential to this angular distribution. The decay parameter for this fit is directly related to the ratio ($\tau$/$\hbox {T}_R$) where $\tau$ is the molecular lifetime against dissociation and $\hbox {T}_R$ is the rotational time period. Thus once the rotational time period of this molecular ion is known, the subrotational lifetime of the metastable state can be extracted.

**Figure 7:**
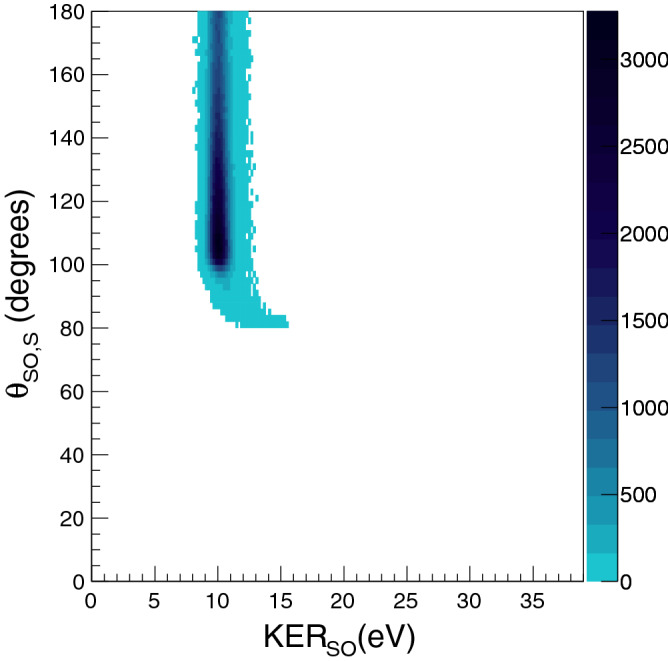
Simulated breakup of $\hbox {SO}_2^{3+}$ into ($\hbox {O}^{+}+\hbox {O}^++\hbox {S}^+$) via sequential mechanism using simple Coulomb explosion model. The rapidly decreasing effect of presence of a nearby neighbor is visible as variation in the $\hbox {KER}_{{SO}}$. See text for details.

Using a value of 16 ps for the rotational period ($\hbox {T}_R$) of $\hbox {SO}^{2+}$ ion, an estimate based on the classical rigid rotor model, and the experimentally determined value of the ratio ($\tau$/$\hbox {T}_R$), we have estimated the lifetime ($\tau$) of its metastable state to be $\approx$2.0 ps with a fitting error of less than 15%. Using these values, we have performed a complete three-body simulation for the sequential breakup of $\hbox {SO}_2^{3+}$ leading to ($\hbox {O}^+ + \hbox {O}^+ + \hbox {S}^+$) using the Coulomb explosion model and calculated the momentum vectors for all three dissociation fragments. For the simulation, we have taken a gaussian distribution of inter-nuclear distances and the bond angle and an exponential decay of the intermediate $\hbox {SO}^{2+}$ with a decay constant being a fraction of its rotational period. Analysing these simulated momenta by using the native frames approach we have generated a distribution of angle $\theta _{SO,S}$ and the kinetic energy released in $\hbox {SO}^{2+}$ breakup, $\hbox {KER}_{{SO}}$. This distribution is shown in Fig. 7. A comparison of Figs. 7 and 6 shows a clear correspondence in the general features of experiment and simulation indicating the existence of subrotational lifetimes. The simplistic Coulomb explosion model is not good enough to give accurate values for the kinetic energy release because of the assumptions entailed in it. Nevertheless, it does highlight the effect of presence of a nearby ion on the unimolecular dissociation of $\hbox {SO}^{2+}$ and hence the need for selection of a subset of $\theta _{SO,S}$ for studying the natural decay of this ion.

## Conclusions

In conclusion, we have measured the subrotational lifetime of a molecular dication by combining two powerful techniques, one experimental viz. coincidence momentum imaging and the other, an analysis procedure, the native frames approach. The lifetime of the $\hbox {SO}^{2+}$ intermediate is determined to be a fraction (1/9) of its rotational period. In the absence of any published report on either a measured or predicted value for the rotational time period of $\hbox {SO}^{2+}$ it is difficult to arrive at an exact value of the lifetime. Nonetheless, taking the approximate value of $\hbox {T}_R$ based on a classical model, the lifetime for $\hbox {SO}^{2+}$ is determined to be $\approx$ 2 ps. To the best of our knowledge, this is the first ever experimental measurement of subrotational metastable lifetimes for molecular dications. This is a proof of principle and may be extended to other diatomic/polyatomic molecular dications whose lifetimes may lie within few picoseconds.

## Data Availability

The data sets pertaining to the current study are available from the corresponding author on reasonable request

## References

[1] Mathur D (2004). Structure and dynamics of molecules in high charge states. Phys. Rep..

[2] Falcinelli S (2016). Molecular dications in planetary atmosphere escape. Atmosphere.

[3] Field TA, Eland JHD (1993). Lifetimes of metastable molecular doubly charged ions. Chem. Phys. Lett..

[4] Šedivcová, T., Žd’ánská, P., Špirko, V. & Fišer, J. Computed lifetimes of metastable states of CO$$^{2+}$$. *J. Chem. Phys.* **124**, 214303 (2006). 10.1063/1.2198835 16774403

[5] Wetmore RW, Boyd RK (1986). Theoretical investigation of the dication of molecular nitrogen. J. Phys. Chem..

[6] Szaflarski DM (1991). Characterization of triplet states in doubly charged positive ions: assignment of the $$^{3}{\Pi }_{g}$$-$$^{3}{\Sigma }_{u}^{+}$$ electronic transition in nitrogen N$$_{2}^{2+}$$. J. Phys. Chem..

[7] Mrugala F (2008). A computational study of metastable states of CO$$^{2+}$$. J. Chem. Phys..

[8] Šedivcova T, Špirko V, Fišer J (2006). Theoretical study of the CS$$^{2+}$$ dication. J. Chem. Phys..

[9] Edvardsson D, Lunell S, Rakowitz F, Marian CM, Karlsson L (1998). Calculation of predissociation rates in O$$_2^{2+}$$ by ab initio MRD-CI methods. Chem. Phys..

[10] Senekowitsch, J., Oeil, S. Metastable $$^{3}{\Sigma }^{-}_{g}$$ ground state of F$$^{++}_{2}$$ and the bonding in molecular dications. *J. Chem. Phys.* **95**, 1847–1851 (1991)

[11] Martin S, Chen L, Al-Mogeeth A, Bernard J (2019). Fragmentation and cooling of doubly charged anthracene studied in an electrostatic storage ring. Phys. Rev. A.

[12] Mathur D, Andersen LH, Hvelplund P, Kella D, Safvan CP (1995). Long-lived, doubly charged diatomic and triatomic molecular ions. J. Phys. B At. Mol. Opt. Phys..

[13] Andersen LH (1993). Very slow spontaneous dissociation of CO$$^{2+}$$ observed by means of a heavy ion storage ring. Phys. Rev. Lett..

[14] Safvan CP, Mathur D (1995). Measurements of dication lifetimes by translational energy spectrometry. Rapid Commun. Mass Spectrom..

[15] Hikosaka Y, Shigemasa E (2019). Metastability of carbonyl sulfide dications studied by multi-electron-ion coincidence spectroscopy. Int. J. Mass Spectrom..

[16] Jochim B (2017). Three-dimensional momentum imaging of dissociation in flight of metastable molecules. New J. Phys..

[17] Sharma V (2007). Dissociative double ionization of CO$$_2$$: Dynamics, energy levels, and lifetime. J. Phys. Chem. A.

[18] Neumann N (2010). Fragmentation dynamics of CO$$_2^{3+}$$ investigated by multiple electron capture in collisions with slow highly charged ions. Phys. Rev. Lett..

[19] Wu C (2013). Nonsequential and sequential fragmentation of CO$$_2^{3+}$$ in intense laser fields. Phys. Rev. Lett..

[20] Rajput J (2018). Native frames: disentangling sequential from concerted three-body fragmentation. Phys. Rev. Lett..

[21] Shen Z, Wang E, Gong M, Shan X, Chen X (2016). Fragmentation dynamics of carbonyl sulfide in collision with 500 eV electron. J. Chem. Phys..

[22] Ramadhan A (2016). Ultrafast molecular dynamics of dissociative ionization in OCS probed by soft x-ray synchrotron radiation. J. Phys. B At. Mol. Opt. Phys..

[23] Kumar H, Bhatt P, Safvan CP, Rajput J (2018). Three-body dissociation of OCS$$^{3+}$$: Separating sequential and concerted pathways. J. Chem. Phys.

[24] Guillemin R (2015). Selecting core-hole localization or delocalization in CS$$_2$$ by photofragmentation dynamics. Nature Comm..

[25] Hishikawa A, Hasegawa H, Yamanouchi K (2002). Sequential three-body coulomb explosion of CS$$_2$$ in intense laser fields appearing in momentum correlation map. Chem. Phys. Lett..

[26] Khan A, Tribedi LC, Misra D (2017). Three-body fragmentation of multiply charged nitrous oxide induced by $$\text{Ar}^{8+}$$- and $${{\rm Xe}}^{15+}$$-ion impact. Phys. Rev. A.

[27] Xu S (2018). Dynamics of $$\text{ C}_2\text{ H}_2^{3+}$$$${\rightarrow }$$$$\text{ H}^+ + \text{ H}^+ + \text{ C}_2^+$$ investigated by 50-kev/u Ne$$^{8+}$$ impact. Phys. Rev. A.

[28] Dalitz RH (1953). On the analysis of $${\tau }$$-meson data and the nature of the $${\tau }$$-meson. Phil. Mag..

[29] Kumar A (2014). Setup for measuring angular anisotropies in slow ion-molecule collisions. Int. J. Mass Spectrom..

[30] Dorner R (2000). Cold target recoil ion momentum spectroscopy: a ‘momentum microscope’ to view atomic collision dynamics. Phys. Rep..

[31] Holder CH, Fink M (1981). Structure determination of SO$$_{2}$$ by electron diffraction. J. Chem. Phys..

[32] Méry A (2017). Role of a neighbor ion in the fragmentation dynamics of covalent molecules. Phys. Rev. Lett..

